# Pheochromocytoma Presenting as Testicular Pain: An Unusual Case Report

**DOI:** 10.1155/2021/6699409

**Published:** 2021-04-14

**Authors:** Jinal K. Patel, Varun Reddy, Gauthier Stepman, Debra Angelo, Johnathan Frunzi

**Affiliations:** Department of Internal Medicine, HCA Healthcare/USF Morsani College of Medicine GME: Medical Center of Trinity, Trinity, FL, USA

## Abstract

Pheochromocytoma (PCC) is a rare catecholamine-secreting tumor that arises from chromaffin cells of the adrenal medulla which are derived from the neural crest. This report illustrates a 51-year-old Caucasian male with a history of hypertension diagnosed two years ago who presented to the hospital due to acute onset of right testicular pain of 3-day duration. Laboratory results and imaging revealed a presumptive diagnosis of PCC. The patient had undergone robot-assisted laparoscopic right adrenalectomy 14 days after being diagnosed with PCC due to perioperative management with phenoxybenzamine. The final pathology report revealed a PCC. At follow-up two weeks after discharge, the patient reported complete resolution of his testicular pain.

## 1. Introduction

Pheochromocytoma (PCC) is a rare catecholamine-secreting tumor that arises from chromaffin cells of the adrenal medulla [1–4]. Histologically, chromaffin cells are derived from the neural crest during development [1–4]. In 2017, the World Health Organization (WHO) classified PCC as an adrenal tumor and extraadrenal tumor as paraganglioma; since both types of tumor cannot be differentiated on histological findings [1–4]. The incidence of PCC and paraganglioma ranges between 2 and 8 per million, whereas the incidence of PCC is approximately 500–1600 cases per year [5]. PCC may occur at any age; however, it is more commonly seen in the 3rd to 5th decade of life [3]. Typically, patients present with a triad of severe headache, sweating, and palpitations which occur in waves as a result of excess hormone release [1–3]. Furthermore, half of the patients can have paroxysmal hypertension. Less common symptoms include flushing, tremor, chest pain, weight loss, constipation, diarrhea, and warmth or heat intolerance [2]. The diagnosis of PCC is a combination of urine and serum analysis showing excess catecholamines and localization of the tumor by computerized tomography (CT) and magnetic resonance imaging (MRI) [1–4, 6]. Here, we present a case of a 51-year-old Caucasian male with PCC presenting as testicular pain.

## 2. Case Presentation

A 51-year-old Caucasian male with a past medical history of hypertension (diagnosed two years ago, treated with lisinopril 10 mg daily), nephrolithiasis, presented to the hospital with a 3-day complaint of right testicular pain. The pain radiated to the right flank, was intermittent, sharp, 5/10 in severity, aggravated with movement, and alleviated with rest. The pain in the scrotum had not improved, which caused him to present to the emergency department. He denied having hypertensive episodes, headaches, sweating, flushing, and palpitations.

**Figure 1:**
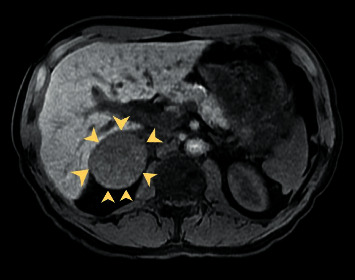
T2-Weighted MRI of the abdomen: slight increased T2 signal and heterogeneous enhancement of the right adrenal gland (arrows).

An initial evaluation in the emergency department revealed the following vitals: temperature of 98.6 F, heart rate of 81 beats per minute, blood pressure (BP) of 137/96 mmHg, and oxygen saturation of 97% on room air. Initial laboratory results revealed mild leukocytosis with a white blood cell (WBC) count of 12,900 cells/mL. Due to the patient having acute scrotal pain and elevated WBC count, testicular etiologies such as testicular torsion, epididymitis, varicocele, hydrocele, or chronic testicular pain were ruled out with a thorough physical examination and testicular ultrasound (US). A CT of the abdomen and pelvis without contrast revealed a large right suprarenal mass. A follow-up CT abdomen with adrenal protocol revealed a 5.1 cm enhancing right adrenal mass, which was further detailed with an MRI with contrast (Figure 1). With findings of an adrenal gland mass on imaging, urine and serum analyses (Tables 1 and 2) were obtained, which revealed elevated catecholamines consistent with possible PCC. The patient was discharged home as he required perioperative blood pressure management with phenoxybenzamine. The patient was brought back to the hospital 14 days later for transperitoneal robot-assisted laparoscopic right adrenalectomy due to having multiple back surgeries. He was transferred to the intensive care unit (ICU) postoperatively for close monitoring of hemodynamics. The final pathology report revealed a PCC, 6.2 cm in size, with no evidence of necrosis, capsular invasion, or extension into periadrenal adipose tissue. The patient was discharged home on postoperative day three and to follow-up with hematology and oncology for surveillance and possible adjuvant chemotherapy. At follow-up two weeks after discharge, the patient reported complete resolution of his testicular pain.

## 3. Discussion

Testicular pain with acute onset warrants emergent evaluation with a thorough history and physical examination as well as urinalysis and imaging [7, 8]. For example, testicular torsion is a medical emergency, which presents as a high-riding testicle, which is confirmed with a scrotal US and can result in loss of testes if there is a delay of care [7–9]. In our patient, we were able to rule out potential medical emergencies with examination, urinalysis, and imaging as stated above. While less common, extrascrotal etiologies can cause testicular pain due to common nerve root pathways (T10-T12, L1-L2, and S2–S4) [10]. Thus, pathologies such as ureteral calculi, back pain, aortic aneurysm, intervertebral disc prolapse, hernias, and other intrabdominal pathologies that share common nerve pathways may present as acute testicular pain [10], which was the case of our patient.

Our case is unique, as there have not been reported cases previously of PCC presenting as testicular pain. On the other hand, Cheungpasitporn et al. reported a case of adrenocortical carcinoma that presented as varicocele and renal vein thrombosis [11].

The majority of PCC are initially discovered as incidentalomas, while others are suspected in patients presenting with the typical symptoms as mentioned in the introduction or secondary cause of hypertension [12]. Our patient likely did not have the typical presentation of PCC as his hypertension was treated with lisinopril for the past 2 years, which likely masked his symptoms of catecholamine excess. In our case, the patient did not have evidence of acute testicular etiology as stated above, which warranted further investigation with a CT of the abdomen and pelvis as the patient had a history of renal calculi. There was no evidence of nephrolithiasis; however, there was an incidental finding of an adrenal mass. When an adrenal mass is found incidentally, the next step in management is to determine if it is malignant or functioning, which can be determined with laboratory tests and imaging studies [1–4]. PCC has increased attenuation on unenhanced CT scan revealing greater than 20 Hounsfield units (HU), with a delay in contrast medium washout [13, 14]. Our patient's CT scan measured 64 HU prior to contrast administration, which enhanced heterogeneously following contrast injection with delay in contrast medium washout. Also, PCC shows high signal intensity on T2-weighted MRI [13, 14]. Our patient's MRI demonstrated a slightly increased T2 signal and heterogeneous enhancement. Due to the imaging findings, PCC was higher on the differential and requires further investigation with biochemical assay [1–6, 15]. A 24-hour urinary metanephrine and plasma metanephrines were positive for excess catecholamines (Tables 1 and 2), further supporting the presumptive diagnosis of PCC. We later obtained a CT of the chest and MRI of the brain for staging, which was negative for metastatic disease. Once the diagnosis of PCC is confirmed, the next step in management is surgical resection [2, 16]. Our patient had undergone robot-assisted laparoscopic right adrenalectomy.

While surgical resection of the tumor is usually definitive, it is associated with high mortality risk if not managed medically first [15]. Physical manipulation of the adrenal gland during surgery can cause a sudden release of catecholamines and cause intraoperative hypertensive emergency [15–17]. Thus, in the perioperative period, it is important to start patients on adrenergic blocking agents 10–14 days prior to surgery to decrease intraoperative hypertensive crisis [15–17]. Initial treatment consists of alpha-blockade with medications like phenoxybenzamine [17]. Our patient in the perioperative period was treated with phenoxybenzamine beginning 14 days prior to surgery. Alpha-blockage prevents peripheral vascular constriction and subsequently causes reflexive tachycardia. Beta-blockers are initially avoided, as blocking this initial reflex can cause dangerous hypertension due to unopposed alpha blockade. Once an adequate alpha-blockade has been achieved, which can be indicated by postural hypotension, a beta-blocker can be added. Our patient did report postural hypotension but was not treated with a beta-blocker. On the other hand, increased salt and fluid intake can be recommended to expand the intravascular volume and maintain BP preoperatively, which was done in our patient. After appropriate perioperative management is achieved, the final step in management is adrenalectomy. A patient may have the option of undergoing an open approach, laparoscopic or robotic-assisted laparoscopy adrenalectomy [16, 18, 19]. Comparing the different surgical approaches, robotic-assisted approach has been shown to decrease blood loss and have shorter hospital course when compared to open and laparoscopic approach [19]. In robotic-assisted adrenalectomy, patients may undergo transperitoneal or retroperitoneal laparoscopic adrenalectomy [16, 18]. Ultimately, our patient had undergone transperitoneal robot-assisted laparoscopic right adrenalectomy as he had multiple previous back surgeries. After surgical removal of the adrenal gland, patients should be monitored closely in the ICU due to potential complications of abrupt decline in catecholamine levels after tumor resection [20]. Finally, patients should be followed up with an MRI for recurrence for at least 10 years even after complete resection [6].

## 4. Conclusion

Physicians should be aware that testicular tenderness needs further evaluation if a thorough history and physical exam do not reveal a cause. Furthermore, the combination of testicular pain and hypertension should raise suspicion for PCC, even in the absence of the typical symptoms for PCC.

## Figures and Tables

**Table 1:** Urine analysis.

Urine analysis	Results	Reference range
Epinephrine	24 ug/L	Undefined
Epinephrine in 24 hours	58	0–20 ug/24 hr
Norepinephrine	128 ug/L	Undefined
Norepinephrine in 24 hours	307	0–135 ug/24 hr
Metanephrine	562 ug/L	Undefined
Metanephrine in 24 hours	1345	45–290 ug/24 hr
Normetanephrine	4520 ug/L	Undefined
Normetanephrine in 24 hours	10848	82–500 ug/24 hr
Dopamine	112 ug/L	Undefined
Dopamine in 24 hours	269	0–510 ug/24 hr

**Table 2:** Serum analysis.

Serum analysis	Results	Reference range
Carcinoembryonic antigen	2.1	0.0–0.5 ng/mL
Renin activity	3.010	0.167–5.380 ng/mL/hr
Aldosterone	1.1	0.0–30.0 ng/dL
DHEA sulfate	151.0	80.0–560.0 ng/dL
AM cortisol	2.29	AM: 4.30–22.40 mcg/dL
PM: 3.09–16.66 mcg/dL		
ACTH	8.9	7.2–63.3 pg/mL
Plasma metanephrine	510	0–62 pg/mL
Plasma normetanephrine	5648	0–145 pg/mL

DHEA: dihydroepiandrosterone, ACTH: adrenocorticotropic hormone.
